# Supplementary material for: Zika virus remodelled ER membranes contain proviral factors involved in redox and methylation pathways
Source: Nat Commun. 2023 Dec 5;14:8045. doi: 10.1038/s41467-023-43665-6 (PMC10698153; doi:10.1038/s41467-023-43665-6)
Supplement: Supplementary file 1 — Supplementary information [file 41467_2023_43665_MOESM1_ESM.pdf]

Supplementary information

Zika virus remodelled ER membranes contain proviral factors involved in redox and methylation pathways

Solene Denolly, Alexey Stukalov, Uladzimir Barayeu, Alina N. Rosinski, Paraskevi Kritsiligkou, Sebastian Joecks, Tobias P. Dick, Andreas Pichlmair, Ralf Bartenschlager

Corresponding author: ralf.bartenschlager@med.uni-heidelberg.de

Supplementary Table 1. siRNA sequences used in this study

Gene Symbol	Catalog Number of siRNA pool	Sequence
ATL2	L-014047-02	GUAGAGAUCUUGUAGAAUA
		UCAAGGAAGUGGCGAUAAA
		GUACUUGGGCAUAUGUUAA
		CCUCCCUAGGUGAGAAUUA
PNPLA8	L-010284-00	UGAAUAACCCUUCGGCAUU
		GAUGUGAGAAACACGGUAA
		CCAAAGCUCUGCAUUACUA
		GCAAGGGUGAGUAUUGAUA
RDX	L-011762-00	GAGCUAAUGGAACGUCUAA
		GGCAUUAAGUUCAGAAUUA
		CUACAUGGCUUAAACUAAA
		GGGCACAAUUAGAGAAUGA
NME2	L-005102-00	GCGAGAUCAUCAAGCGCUU
		GAAAUCAGCCUAUGGUUUA
		AAUAAGAGGUGGACACAAC
		CUGAAGAACACCUGAAGCA
TXNRD1	L-008236-00	GCAUCAAGUUUAUAAGACA
		GCGAUAUAUUGGAGGAUAA
		CUAAGGAGGCAGCCCAAUA
		GGACAGCACAAUUGGAAUC
AKR1C3	L-008116-00	CCACCCUAAUUAUCCAUAU
		CGGAGUAAAUUGCUAGAUU
		AGACAGAAAUCUCCACUAU
		CGAAAGAUAUUGUUCUGGU
SERPINH1	L-011230-00	CAAAGCGGCUCCCUGCUAU
		GCACUGCGGAGAAGUUGAG
		AAGAUCAACUUCCGCGACA
		AGAAACACCUGGCUGGGCU
SOD1	L-008364-00	GGAAGUCGUUUGGCUUGUG
		GCACACUGGUGGUCCAUGA
		GUGCAGGGCAUCAUCAAUU
		CAAUAAACAUUCCCUUGGA
BLVRB	L-004040-02	GCAAGCAGGUUACGAAGUG
		CAAGGGUCAUCUCCAAACA
		CCGAUGAGUACGACGGACA
		CACAAGGUGCUGCGGGAAU
PGD	L-008371-00	GAAUAUAGGGACACCACAA
		GAUCAUCUCUUACGCUCAA
		CGAGAAAUUGGUACCAUUG
		GAGCAGGCCACUUCGUGAA
SCD	L-005061-00	CUACAAGAGUGGCUGAGUU
		CUACGGCUCUUUCUGAUCA
		GCACAUCAACUUCACCACA

		GAACAGUGCUGCCCACCUC
TPI1	L-009776-00	GAGCCUGUGUGGGCCAUUG
		CCAGGAAGUACACGAGAAG
		GGGUGGUGCUUCCCUCAAG
		GCAGAAAGUGGCCCAUGCU
AHCY	L-009599-00	GGACCCAUCCAGACAAGUA
		GCGAAACGGACGAGGAGUA
		CAAGGUGCCUGCCAUCAAU
		GCAUUGAGCAGACCCUGUA
PGK1	L-006767-00	GGACAAGCUGGACGUUAAA
		GGGCGGAGCUAAAGUUGCA
		GAACAAGGUUAAAGCCGAG
		GAGCUGAACUACUUUGCAA
ON-TARGETplus Non-targeting Control	D-001810-10	UGGUUUACAUGUCGACUAA
PDE3B	L-007646-00	CAGGAAGGAUUCUCAGUCA
		GUAAAGCUGAUGGGAAUAA
		GCGAAUUGCUUAUAUUUCU
		GGCAUAGAAUGGAGUAAUG
PDE2A	L-007644-00	GGAAAGUCCGGGAGGCUAU
		CGACGGCCUUCUCCAUCUA
		CCCAUUCUCUCCUAUACAA
		GGCCAUGGUUCAGCAAGUU

Supplementary Table 2. Primers used in this study for qPCR

	Forward	Reverse
HPRT	CCTGGCGTCGTGATTAGTG	ACACCCTTTCCAAATCCTCAG
ZIKV	AGATGAACTGATTGGCCGGGC	AGGTCTCTTCTGTGGAAATA
ZIKV (-) strand	GGCCGTCATGGTGGCGAATAA	CCTGACAACACTAAGATTGGTGC
HCV	TCTGCGGAACCGGTGAGTA	GGGCAT AGAGTGGGTTTATCCA
PNPLA8	GCCCTAATTGGCTATGTGGATCC	TTCGTAGGGTCTGGAGAGCAAC
RDX	GAAAATGCCGAAACCAATCAA	GTATTGGGCTGAATGGCAAATT
NME2	AAGCAGCACTACATTGACCTGAAA	GGTCTCCCCAAGCATCACTC
TXNRD1	GTTACTTGGGCATCCCTGGTGA	CGCACTCCAAAGCGACATAGGA
AKR1C3	GAGAAGTAAAGCTTTGGAGGTCACA	CAACCTGCTCCTCATTATTGTATAAATGA
SERPINH1	AGGTCACCAAGGATGTGGA	CAGCTTCTCCTTCTCGTCGT
SOD1	GGTCCTCACTTTAATCCTCTATCCAG	CCAACATGCCTCTCTTCATCC
BLVRB	CTCATGGTGTGGACAAGGTCGT	CATCACAGCCACGTACTTCAGG
PGD	GTTCCAAGACACCGATGGCAAAC	CACCGAGCAAAGACAGCTTCTC
SCD	TCTAGCTCCTATACCACCACCA	TCGTCTCCAACTTATCTCCTCC
TPI1	CGCAGATAACGTGAAGGAC	CAGTCACAGAGCCTCCATAA
AHCY	ATCCTTGGCCGGCACTTTGAG	TCCACCTGCGGCTTGATGTTC
PGK1	CAAGAAGTATGCTGAGGCTGTCA	CAAATACCCCCACAGGACCAT
ACTB	TGACAAAACCTAACTTGCGCAG	TCACCTTCACCGTTCCAGTTTT
G3BP2	AAGGACATGCCCACTGAATTCA	CCCAGCATTAACAGGCAGAAAC

Supplementary Table 3. Antibodies used for Western Blot or IF.

Primary antibodies		
**Targets**	**Dilution**	**Company (catalog number)**
GAPDH	WB: 1:1000	Santa Cruz Biotechnology (Cat #sc-365062)
Calnexin	WB: 1:1000	Enzo life sciences (Cat #ADI-SPA-860-F)
HA (mouse)	IF: 1:200	Sigma-Aldrich (H3663-200UL)
HA (rabbit)	IF: 1:200; WB:1:1000	ThermoFisher (PA1-985)
AKR1C3	IF: 1:100; WB: 1:1000	R&D Systems (MAB7678)
GM130	WB:1:1000	BD (610823)
BLVRB	IF: 1:100; WB: 1:1000	Sigma-Aldrich (HPA041698-25UL)
AHCY	IF: 1:100; WB: 1:2000	Sigma-Aldrich (HPA044675-25UL)
TXNRD1	IF:1:100; WB: 1:1000	R&D Systems (MAB7428)
actin	WB: 1:10000	Sigma-Aldrich (AC-74)
apoE	WB: 1:500	Sigma-Aldrich (AB947)
Alpha-1-anti-trypsin	WB: 1:1000	Sigma-Aldrich (A0409-1VL)
Lamp1	IF: 1:200	ThermoFisher (14-1079-80)
dsRNA	IF: 1:250	SCICONS (10010200)
ZIKV capsid	IF: 1:200; WB:1:1000	Genetex (GTX133317)
ZIKV prM	WB:1:1000	Genetex (GTX133584)
ZIKV Env	WB:1:1000	Genetex (GTX133325)
ZIKV Env	WB:1:10	panFlavi (4G2) hybridoma
ZIKV NS1	WB:1:1000	Genetex(GTX5212)
ZIKV NS2B	WB:1:1000	Genetex GTX133318
ZIKV NS3	WB:1:1000	Genetex (GTX133320)
ZIKV NS4A	WB:1:1000	Genetex(GTX133704)
ZIKV NS4B	WB:1:1000	Genetex(GTX133321)
ZIKV NS5	WB:1:1000	Genetex (GTX133327)
**Secondary antibodies**		
Goat anti-rabbit IgG HRP	WB:1:4000	Sigma Aldrich (A6154)
Goat anti-mouse IgG HRP	WB:1:4000	Sigma Aldrich (A4416)
Rabbit anti-goat IgG HRP	WB:1:2000	Sigma Aldrich (A5420)
Donkey anti-Rabbit IgG (H+L) Alexa Fluor 488	IF: 1:1000	ThermoFisher (A21206)
Donkey anti-Mouse IgG (H+L) Alexa Fluor 488	IF: 1:1000	ThermoFisher (A21202)
Donkey anti-Mouse IgG (H+L) Alexa Fluor 568	IF: 1:1000	ThermoFisher (A10037)
Donkey anti-Rabbit IgG (H+L) Alexa Fluor 568	IF: 1:1000	ThermoFisher (A10042)
Donkey anti-Rabbit IgG (H+L) Alexa Fluor 647	IF: 1:1000	ThermoFisher (A31573)
Goat anti-Mouse IgG1 Alexa Fluor 647	IF: 1:1000	ThermoFisher (A21240)
Goat anti-Mouse IgG1 Alexa Fluor 488	IF: 1:1000	ThermoFisher (A21121)
Goat anti-Mouse IgG2a Alexa Fluor 568	IF: 1:1000	ThermoFisher (A21134)

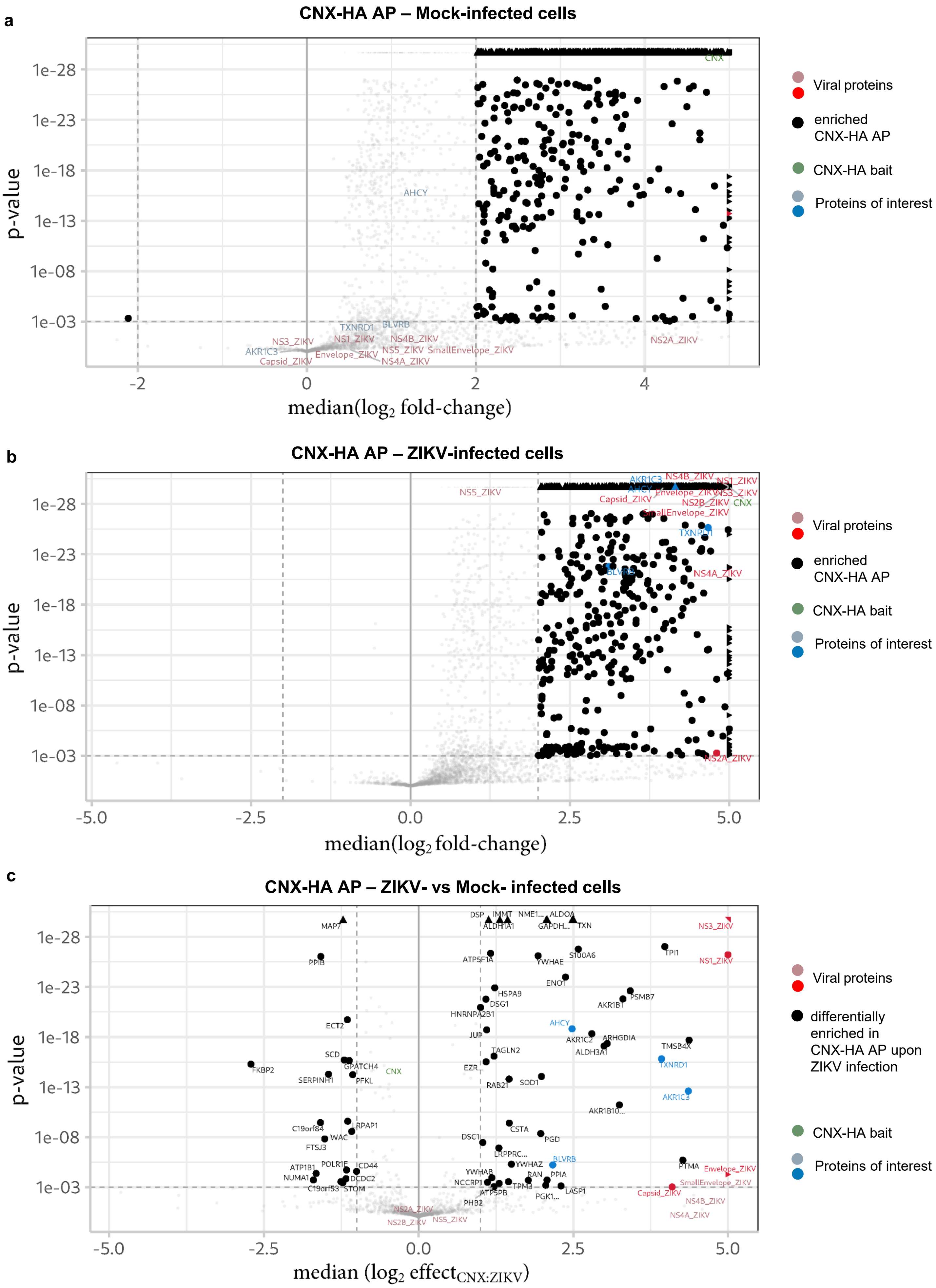
a
CNX-HA AP – Mock-infected cells
p-value
1e-28
1e-23
1e-18
1e-13
1e-08
1e-03
-2
0
2
4
median(log2 fold-change)
CNX
AHCY
TXNRD1
BLVRB
NS3_ZIKV
NS1_ZIKV
NS4B_ZIKV
AKR1C3
Envelope_ZIKV
NS5_ZIKV
SmallEnvelope_ZIKV
Capsid_ZIKV
NS4A_ZIKV
NS2A_ZIKV
Viral proteins
enriched CNX-HA AP
CNX-HA bait
Proteins of interest
b
CNX-HA AP – ZIKV-infected cells
p-value
1e-28
1e-23
1e-18
1e-13
1e-08
1e-03
-5.0
-2.5
0.0
2.5
5.0
median(log2 fold-change)
NS5_ZIKV
AKR1C3
NS4B_ZIKV
NS1_ZIKV
AHCY
Envelope_ZIKV
NS3_ZIKV
Capsid_ZIKV
NS2B_ZIKV
CNX
SmallEnvelope_ZIKV
TXNRD1
BLVRB
NS4A_ZIKV
NS2A_ZIKV
Viral proteins
enriched CNX-HA AP
CNX-HA bait
Proteins of interest
c
CNX-HA AP – ZIKV- vs Mock- infected cells
p-value
1e-28
1e-23
1e-18
1e-13
1e-08
1e-03
-5.0
-2.5
0.0
2.5
5.0
median (log2 effectCNX:ZIKV)
MAP7
DSP
IMMT
NME1...
ALDOA
ALDH1A1
GAPDH...
TXN
NS3_ZIKV
PPIB
ATP5F1A
YWHAE
S100A6
TPI1
NS1_ZIKV
ENO1
HSPA9
PSMB7
DSG1
AKR1B1
HNRNPA2B1
ECT2
AHCY
JUP
AKR1C2
ARHGDIA
ALDH3A1
TMSB4X
SCD
TAGLN2
GPATCH4
CNX
EZR...
TXNRD1
FKBP2
SERPINH1
PFKL
RAB21
SOD1
AKR1C3
AKR1B10...
C19orf84
LRPAP1
CSTA
WAC
DSC1
PGD
FTSJ3
LRPPRC...
ATP1B1
POLR1E
CD44
YWHAB
YWHAZ
BLVRB
PTMA
Envelope_ZIKV
NUMA1
DCDC2
NCCRP1
RAN
PPIA
SmallEnvelope_ZIKV
C19orf53
STOM
TPM3
ATP5PB
LASP1
Capsid_ZIKV
PGK1...
NS4B_ZIKV
PHB2
NS2A_ZIKV
NS4A_ZIKV
NS2B_ZIKV
NS5_ZIKV
Viral proteins
differentially enriched in CNX-HA AP upon ZIKV infection
CNX-HA bait
Proteins of interest

Supplementary Figure 1. Statistical analysis of ZIKV-induced changes in protein enrichment upon CNX-HA affinity purification. Viral proteins are highlighted in red; proteins characterized further in blue; CNX-HA bait in green. Light colors were used to highlight proteins without enrichment. **(a)** Enrichment of proteins upon CNX-HA affinity purification (AP) from lysates of Mock-infected A549 cells. The X axis is the median of the posterior distribution of $\log_2$ fold-change between protein intensity upon CNX-HA AP and empty bait AP control, both obtained with Mock-infected A549 cells. The Y axis is the p-value (two-sided, based on the Bayesian linear model, unadjusted, n=6 biological batches). Larger dots denote significantly enriched proteins (median ($\log_2$ *fold-change*) ≥ 2, p-value ≤ 1E-3). **(b)** Enrichment of proteins upon CNX-HA AP from lysates of ZIKV-infected A549 cells. The X axis is the median of the posterior distribution of log2 fold-change between protein intensity upon CNX-HA AP and empty bait AP control, both obtained with ZIKV-infected A549 cells. The Y axis is the p-value (two-sided, based on the Bayesian linear model, unadjusted, n=6 biological batches). Larger dots denote significantly enriched proteins (median ($\log_2$ *fold-change*) ≥ 2, p-value ≤ 1E-3). **(c)** Changes of protein enrichment in CNX-HA AP induced by ZIKV infection of A549 cells. The X axis is the median of the posterior distribution of the model CNX:ZIKV effect that corresponds to the following linear combination of protein intensities:

$$effect_{CNX:ZikV} = \left(\log_2 I_{CNX,ZikV} - \log_2 I_{CNX,Mock}\right) - \left(\log_2 I_{empty,ZikV} - \log_2 I_{empty,Mock}\right).$$

The Y axis is the p-value (two-sided, based on the Bayesian linear model, unadjusted, n=6 biological batches). Larger dots denote significantly enriched proteins (|median ($\log_2$ *fold-change*) | ≥ 1, p-value ≤ 1E-3). For a complete list of enriched proteins, see Supplementary Data 1.

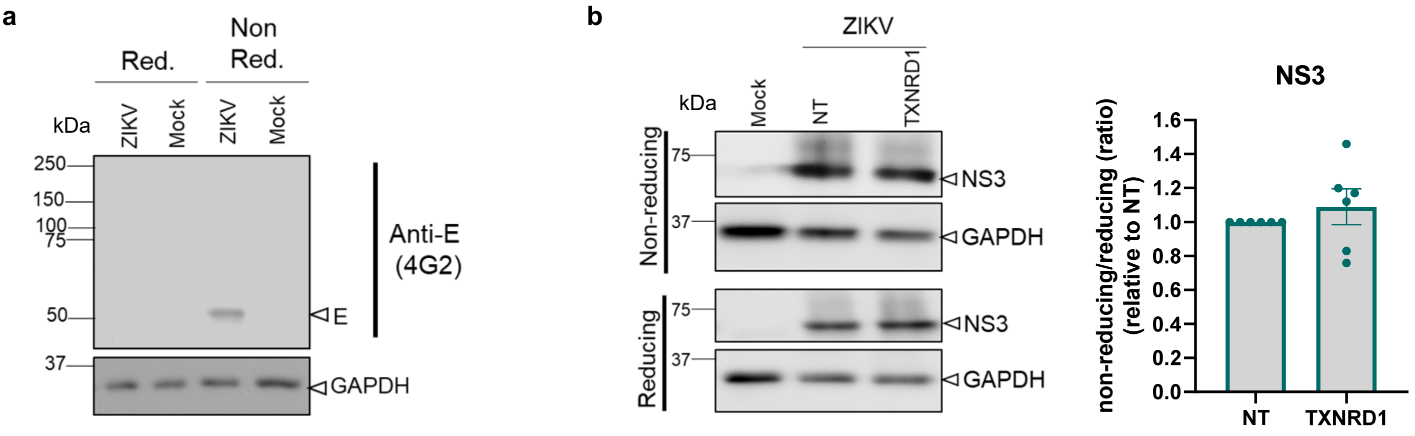

Supplementary Figure 2. Role of TXNRD1 in ZIKV particle secretion. (a) Lysates of Mock and ZIKV-infected cells were analyzed by western blot after SDS-PAGE under reducing or non-reducing conditions. Blots were probed with the E-specific monoclonal antibody 4G2; GAPDH served as loading control. n=3 **(b)** Lysates of A549 cells shown in Figure 4f were analyzed the same way, but using NS3-specific antibody (mean ± SEM of 6 independent experiments).

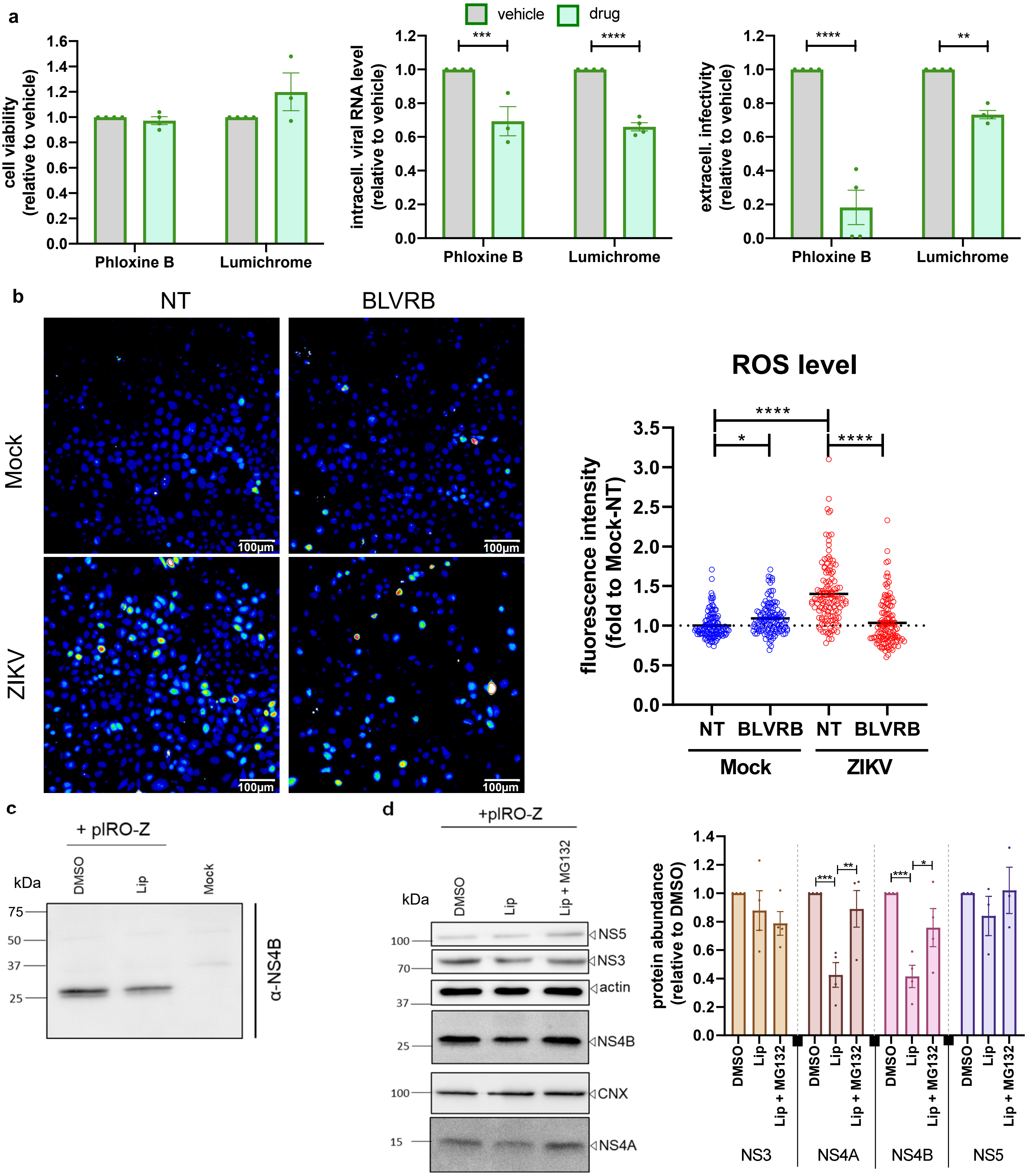
a
vehicle
drug
cell viability (relative to vehicle)
1.6
1.4
1.2
1.0
0.8
0.6
0.4
0.2
0.0
Phloxine B
Lumichrome
intracell. viral RNA level (relative to vehicle)
***
****
extracell. infectivity (relative to vehicle)
****
**
b
NT
BLVRB
Mock
ZIKV
100µm
ROS level
fluorescence intensity (fold to Mock-NT)
3.5
3.0
2.5
2.0
1.5
1.0
0.5
0.0
*
****
NT
BLVRB
Mock
ZIKV
c
+ pIRO-Z
kDa
DMSO
Lip
Mock
75
50
37
25
α-NS4B
d
+pIRO-Z
kDa
DMSO
Lip
Lip + MG132
100
70
37
25
100
15
NS5
NS3
actin
NS4B
CNX
NS4A
protein abundance (relative to DMSO)
1.4
1.2
1.0
0.8
0.6
0.4
0.2
0.0
***
**
*
NS3
NS4A
NS4B
NS5

Supplementary Figure 3. Role of BLVRB in ZIKV replication. (a) A549 cells were infected with ZIKV (MOI=3) and then treated with Phloxine B (10 µM) or Lumichrome (50 µM)**.** Cell viability was assessed with non-infected cells (left) (mean ± SEM, n=3), Intracellular viral RNA (middle) (mean ± SEM, n=3-4, 2way Anova Sidak's multiple comparisons test (vehicle vs. drug), p=0.0002 (Phloxine B), p<0.0001 (lumichrome)). Titers of infectious extracellular virus (right) (mean ± SEM, n=3-4, , 2way Anova Sidak's multiple comparisons test (vehicle vs. drug), p<0.0001 (Phloxine B), p=0.0073 (lumichrome)). **(b)** ROS in A549 cells transfected with BLVRB-targeting or NT siRNAs for 48 h and infected with ZIKV for 24 h was visualized by using DCFDA**.** Representative images (left). Scale bars represent 100µm. Quantification of fluorescent intensity of 40 cells /experiments (right). Values were normalized to those of Mock-DMSO cells(mean ± SEM, n=3-4, one-way ANOVA with Sidak's multiple comparisons test, p=0.0339 (Mock :NT vs. BLVRB), p<0.0001 (NT: Mock vs. ZIKV and ZIKV: NT vs. BLVRB)). **(c)** Samples presented in Figure 6c were analyzed for NS4B precursors. n=3 **(d)** Huh7-Lunet/T7 cells were transfected with a plasmid encoding ZIKV NS1-NS5 (pIRO-Z) and treated with DMSO or Liproxstatin-1 (10 µM) ± MG132 (0.5 µM) 4 h post transfection. Cells were lysed 19 h post transfection and lysates were analyzed by western blot (left panel). Signal intensities were quantified and normalized to CNX or actin loading control (right panel). Values were normalized to DMSO control (mean ± SEM, n=3-5, 2way ANOVA with Tukey's multiple comparisons test; NS4A: DMSO vs. Lip : p=0.0004, Lip vs. Lip+MG132: p=0.0038 ; NS4B: DMSO vs. Lip : p=0.0003, Lip vs. Lip+MG132: p=0.038).

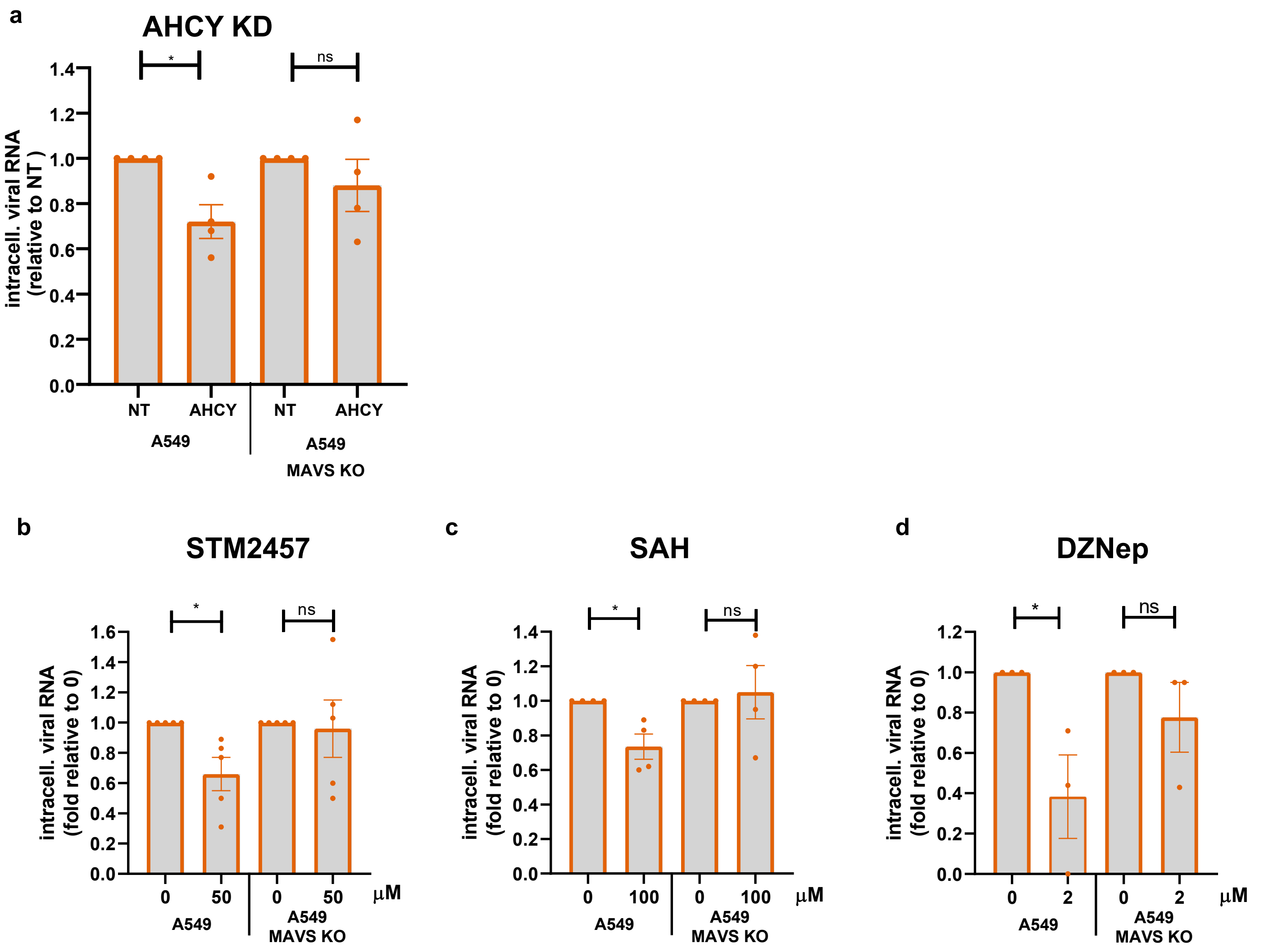

Supplementary Figure 4. Role of AHCY in ZIKV replication. (a) Intracellular viral RNA in samples presented in Figure 8f was quantified by RT-qPCR (mean ± SEM, n=4, Kruskal-Wallis test with Dunn's multiple comparison test (NT vs. AHCY), p=0.0223 (A549), p=0,4088 (A549-MAVS KO)). **(b)** Intracellular viral RNA of samples presented in Figure 8i was assessed by RT-qPCR (mean ± SEM of 5, Kruskal-Wallis test with Dunn's multiple comparison test (0 vs. 50), p=0.0383 (A549), p>0,999 (A549-MAVS KO)). **(c)** Intracellular viral RNA of samples presented in Figure 8j was quantified by RT-qPCR (mean ± SEM, n=4, Kruskal-Wallis test with Dunn's multiple comparison test (0 vs. 100), p=0.0347 (A549), p>0,999 (A549-MAVS KO)). **(d)** Intracellular viral RNA of samples presented in Figure 8k was quantified by RT-qPCR (mean ± SEM, n=4, Kruskal-Wallis test with Dunn's multiple comparison test (0 vs. 2), p=0.0261 (A549), p>0,121 (A549-MAVS KO)).

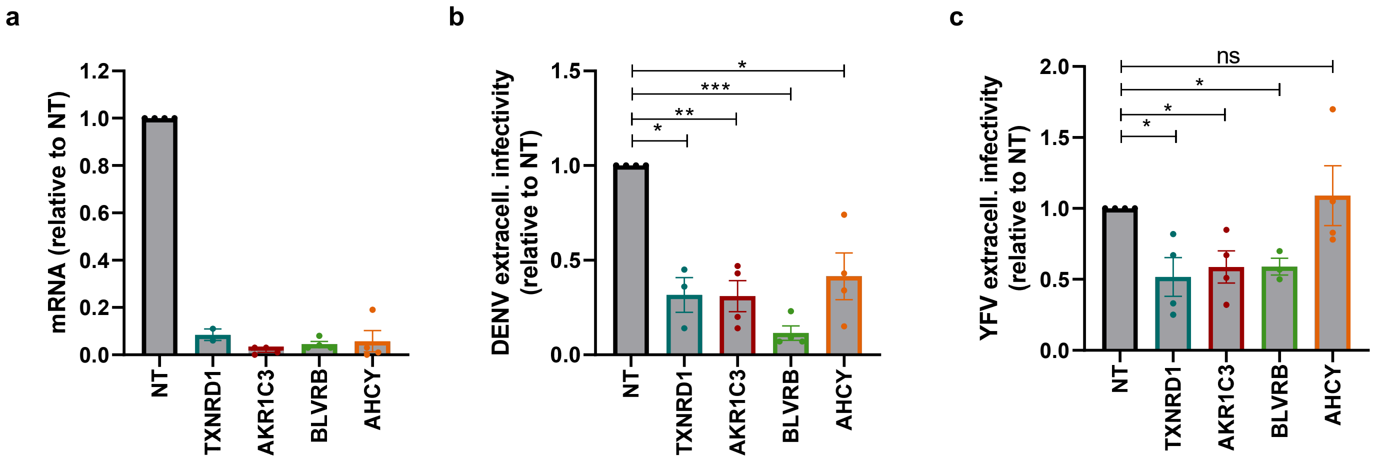

Supplementary Figure 5. Role of the ZIKV dependency factors TXNRD1, AKR1C3, BLVLRB and AHCY in the life cycle of DENV and YFV. A549 cells were reverse transfected with given siRNAs and 48 h later, KD efficiency was assessed by RT-qPCR **(a)**. Cells were infected with DENV (MOI=3) **(b)** or YFV (MOI=0.1). **(c)** Supernatants were harvested 24 h post infection and used to measure production of infectious virus particles by plaque assay. mean ± SEM, n=3-4, one-sample t test (two-tailed). DENV : TXNRD1 p=0.0177, AKR1C3 p=0.0035, BLVRB p=0.0002, AHCY p=0.0177; YFV: TXNRD1 p=0.038, AKR1C3 p=0.0355, BLVRB p=0.0198, AHCY p=0.6993.
