# Supplementary material for: Zika virus remodelled ER membranes contain proviral factors involved in redox and methylation pathways
Source: Nat Commun. 2023 Dec 5;14:8045. doi: 10.1038/s41467-023-43665-6 (PMC10698153; doi:10.1038/s41467-023-43665-6)
Supplement: Supplementary file 3 — Description of Additional Supplementary Files [file 41467_2023_43665_MOESM3_ESM.docx]

**Description of additional supplementary files**

**Title:** Supplementary Data 1

**Description:**

This data set corresponds to the statistical analysis of protein enrichment upon CANX-HA affinity purification in Mock- and ZIKV-infected cells. The data set was used to generate Figure 2a.
