# Supplementary material for: Zika virus remodelled ER membranes contain proviral factors involved in redox and methylation pathways
Source: Nat Commun. 2023 Dec 5;14:8045. doi: 10.1038/s41467-023-43665-6 (PMC10698153; doi:10.1038/s41467-023-43665-6)
Supplement: Supplementary file 5 — Reporting Summary [file 41467_2023_43665_MOESM5_ESM.pdf]

nature portfolio

Corresponding author(s): R. Bartenschlager

Last updated by author(s): November 6th 2023

Reporting Summary

Nature Portfolio wishes to improve the reproducibility of the work that we publish. This form provides structure for consistency and transparency in reporting. For further information on Nature Portfolio policies, see our Editorial Policies and the Editorial Policy Checklist.

Statistics

For all statistical analyses, confirm that the following items are present in the figure legend, table legend, main text, or Methods section.

n/a	Confirmed	
☐	☒	The exact sample size (*n*) for each experimental group/condition, given as a discrete number and unit of measurement
☐	☒	A statement on whether measurements were taken from distinct samples or whether the same sample was measured repeatedly
☐	☒	The statistical test(s) used AND whether they are one- or two-sided<br>*Only common tests should be described solely by name; describe more complex techniques in the Methods section.*
☒	☐	A description of all covariates tested
☐	☒	A description of any assumptions or corrections, such as tests of normality and adjustment for multiple comparisons
☐	☒	A full description of the statistical parameters including central tendency (e.g. means) or other basic estimates (e.g. regression coefficient) AND variation (e.g. standard deviation) or associated estimates of uncertainty (e.g. confidence intervals)
☐	☒	For null hypothesis testing, the test statistic (e.g. *F*, *t*, *r*) with confidence intervals, effect sizes, degrees of freedom and *P* value noted<br>*Give P values as exact values whenever suitable.*
☐	☒	For Bayesian analysis, information on the choice of priors and Markov chain Monte Carlo settings
☒	☐	For hierarchical and complex designs, identification of the appropriate level for tests and full reporting of outcomes
☒	☐	Estimates of effect sizes (e.g. Cohen's *d*, Pearson's *r*), indicating how they were calculated

Our web collection on statistics for biologists contains articles on many of the points above.

Software and code

Policy information about availability of computer code

Data collection	- Immunofluoresence image data were collected using Zeiss Cell Discover 7, Leica SP8, Zeiss AiryScan 2 LSM900 , or Nikon Eclipse Ti microscopes.<br>- western blot data were collected using an INTAS chemocam imager (chemostar software package)<br>- qPCR data were obtained using CFX96 Real Time System from Biorad<br>- cell viability data were obtained using the plate reader Mitras LB 940 from Berthold Technologies Bioanalytics<br>- luciferase data were obtained using the Tube Luminometer (Lumat LB) from Berthold Technologies Bioanalytics<br>- MS data were processed using MaxQuant (version 2.0.3.1)
Data analysis	- Immunofluoresence images were analyzed with the FIJI software package<br>- western blot images were analyzed with the FIJI software package<br>- qPCR data were analyzed using the CFX Maestro V2 program from Biorad<br>- for MS analysis: R (v4.1) and msglm v0.5 (https://github.com/innatelab/msglm/releases/tag/v0.5.0), Stan (v2.29)<br>- The specific scripts for the analysis of our data were deposited to Zenodo (https:/doi.org/10.5281/zenodo.8381245)<br>- GraphPad Prism software (version 8) was used for plotting the results and statistical tests

For manuscripts utilizing custom algorithms or software that are central to the research but not yet described in published literature, software must be made available to editors and reviewers. We strongly encourage code deposition in a community repository (e.g. GitHub). See the Nature Portfolio guidelines for submitting code & software for further information.

Data

Policy information about availability of data

All manuscripts must include a data availability statement. This statement should provide the following information, where applicable:

- Accession codes, unique identifiers, or web links for publicly available datasets
- A description of any restrictions on data availability
- For clinical datasets or third party data, please ensure that the statement adheres to our policy

Source data are provided with the paper as Source Data file.
The mass spectrometry data have been deposited at the ProteomeXchange Consortium via the PRIDE partner repository with the dataset identifier PXD043372.
The scripts for data processing/analysis have been deposited at Zenodo (https:/doi.org/10.5281/zenodo.8381245, https://doi.org/10.5281/zenodo.7746897, https:/doi.org/10.5281/zenodo.7752068)

Research involving human participants, their data, or biological material

Policy information about studies with human participants or human data. See also policy information about sex, gender (identity/presentation), and sexual orientation and race, ethnicity and racism.

Reporting on sex and gender	NA
Reporting on race, ethnicity, or other socially relevant groupings	NA
Population characteristics	NA
Recruitment	NA
Ethics oversight	NA

Note that full information on the approval of the study protocol must also be provided in the manuscript.

Field-specific reporting

Please select the one below that is the best fit for your research. If you are not sure, read the appropriate sections before making your selection.

[x] Life sciences [ ] Behavioural & social sciences [ ] Ecological, evolutionary & environmental sciences

For a reference copy of the document with all sections, see nature.com/documents/nr-reporting-summary-flat.pdf

Life sciences study design

All studies must disclose on these points even when the disclosure is negative.

Sample size	No sample size calculation was performed. Our experiments involved infection in cultured cells where sample size is not relevant. We performed experiments with at least 50,000 cells per infection experiment. Details to that are given in the publication.
Data exclusions	We did not exclude any data in any of the experiments performed in cell culure.
Replication	A minimum of three biological experiments were performed independently on separate days, using separate batches of viruses and different passages of cell lines to confirm reproducibility of the phenotypes. The exact numbers of biological replicates are indicated in the figure legends.
Randomization	No randomization was used as our experiments only involved cell culture experiments. Random sampling for IF images was done and the number of cells is indicated in the figures legends.
Blinding	No blinding was done. Analyses were conducted in an automated manner using software reducing the occurence of bias.

Reporting for specific materials, systems and methods

We require information from authors about some types of materials, experimental systems and methods used in many studies. Here, indicate whether each material, system or method listed is relevant to your study. If you are not sure if a list item applies to your research, read the appropriate section before selecting a response.

Materials & experimental systems

n/a	Involved in the study
	☒ Antibodies
	☒ Eukaryotic cell lines
☒	Palaeontology and archaeology
☒	Animals and other organisms
☒	Clinical data
☒	Dual use research of concern
☒	Plants

Methods

n/a	Involved in the study
☒	ChIP-seq
☒	Flow cytometry
☒	MRI-based neuroimaging

Antibodies

Antibodies used

anti GAPDH mouse Santa Cruz Biotechnology (Cat #sc-365062) WB 1:1000
anti Calnexin rabbit Enzo life sciences (Cat #ADI-SPA-860-F) WB 1:1000
anti HA mouse Sigma-Aldrich (H3663-200UL) IF 1:200
anti HA rabbit ThermoFisher (PA1-985) IF: 1:200; WB:1:1000
anti AKR1C3 mouse R&D Systems (MAB7678) IF: 1:100; WB:1:1000
anti GM130 mouse BD (610823) WB:1:1000
anti BLVRB rabbit Sigma-Aldrich (HPA041698-25UL) IF: 1:100; WB:1:1000
anti AHCY rabbit Sigma-Aldrich (HPA044675-25UL) IF: 1:100; WB:1:1000
anti TXNRD1 mouse R&D Systems (MAB7428) IF: 1:100; WB:1:1000
anti dsRNA mouse SCICONS (10010200) IF: 1:250
anti ZIKV capsid Genetex (GTX133317) WB: 1:1000
anti ZIKV prM Genetex (GTX133584) WB: 1:1000
anti ZIKV Env Genetex (GTX133325) WB: 1:1000
anti ZIKV Env pan flavi (4G2) home-made hybridoma WB: 1:10
anti ZIKV NS1 Genetex(GTX5212) WB: 1:1000
anti ZIKV NS2B Genetex (GTX133318) WB: 1:1000
anti ZIKV NS3 Genetex (GTX133320) WB: 1:1000
anti ZIKV NS4A Genetex(GTX133704) WB: 1:1000
anti ZIKV NS4B Genetex(GTX133321) WB: 1:1000
anti ZIKV NS5 Genetex (GTX133327) WB: 1:1000
anti Lamp1 ThermoFisher (14-1079-80) IF 1:200
anti alpha-1-anti-trypsin Sigma-Aldrich (A0409-1VL) WB: 1:1000
anti actin Sigma-Aldrich (AC-74) WB: 1:10000
anti apoE Sigma-Aldrich (AB947) WB: 1:500

goat anti rabbit IgG HRP Sigma Aldrich(A6154) WB 1:4000
goat anti mouse IgG HRP Sigma Aldrich(A4416) WB 1:4000
rabbit anti goat IgG HRP Sigma Aldrich(A5420) WB 1:2000
goat anti rabbit IgG HRP Sigma Aldrich(A6154) WB 1:4000
Donkey anti-Rabbit IgG (H+L) Alexa Fluor 488 ThermoFisher (A21206) IF 1:1000
Donkey anti-Mouse IgG (H+L) Alexa Fluor 488 ThermoFisher (A21202) IF 1:1000
Donkey anti-Mouse IgG (H+L) Alexa Fluor 568 ThermoFisher (A10037) IF 1:1000
Donkey anti-Rabbit IgG (H+L) Alexa Fluor 568 ThermoFisher (A10042) IF 1:1000
Donkey anti-Rabbit IgG (H+L) Alexa Fluor 647 ThermoFisher (A31573) IF 1:1000
Goat anti-Mouse IgG1 Alexa Fluor 647 ThermoFisher (A21240) IF 1:1000
Goat anti-Mouse IgG1 Alexa Fluor 488 ThermoFisher (A21121) IF 1:1000
Goat anti-Mouse IgG2a Alexa Fluor 568 ThermoFisher (A21134) IF 1:1000

Validation

Antibodies targeting ZIKV proteins were validated by analyzing Mock samples in parallel (both in WB and IF).
Antibodies targeting the HA tag were validated by running samples containing homologous non-tagged proteins in parallel (both in WB and IF).
Antibodies targeting TXNRD1, AHCY, BLVRB and AKR1C3 were validated by analyzing in parallel samples transfected with siRNAs targeting the corresponding gene.
Commercial antibodies were used according to manufacturer's recommendation and validated applications.

Eukaryotic cell lines

Policy information about cell lines and Sex and Gender in Research

Cell line source(s)

A549(ATCC, CCL-185)
HEK293T (ATCC, CCL-3216)
C6/36 (ATCC, CRL 1660),
VeroE6 (ATCC, CRL 1586)

	Huh7-lunet/T7 cells have been described in Appel et al., 2005 doi: 10.1128/JVI.79.5.3187-3194<br>A549-CNX-HA generated in this study<br>A549-MAVS KO cells have been described in Plociennikowska et al., 2021 doi:10.1128/JVI.01050-20
Authentication	None of the cell lines was authenticated beyond visual inspection.
Mycoplasma contamination	All the cell lines were regularly tested and found negative for mycoplasma.
Commonly misidentified lines<br>(See ICLAC register)	No cell lines from the ICLAC register.
